# The current utilization and perceptions of prescription drug monitoring programs among emergency medicine providers in Florida

**DOI:** 10.1186/s12245-017-0140-0

**Published:** 2017-04-18

**Authors:** Henry W. Young, Joseph A. Tyndall, Linda B. Cottler

**Affiliations:** 1 Department of Emergency Medicine, University of Florida, 1329 SW 16th Street, PO Box 100186, Gainesville, FL 32610-0186 USA; 2 Department of Epidemiology, University of Florida, 2004 Mowry Rd, PO Box 100231, Gainesville, FL 32610-0231 USA

**Keywords:** Prescription drug monitoring program, Opioid prescribing, Emergency department

## Abstract

**Background:**

Pain is among the most commonly treated symptoms in the emergency department, and opioids are commonly prescribed from the emergency department to treat moderate to severe pain. Prescription drug monitoring programs (PDMP) can be used to assist physicians identify individuals at increased risk to misuse or abuse opioids. While the use of the PDMP has been shown useful among clinicians, in the past, utilization of the PDMP has been less than optimal. The objective of this study was to assess the current utilization and perceptions of the prescription drug monitoring program among emergency medicine providers in Florida.

**Methods:**

A survey assessing the utilization and perception of Florida’s prescription drug monitoring program was distributed to emergency medicine providers in Florida over a 5 week period. Attending physicians, physicians in training, and extenders from a variety of practice types were assessed.

**Results:**

A total of 88 surveys were completed. Over two thirds (67%) of the respondents were male. The majority of respondents were attending physicians (62%), 13 (14%) were residents, and 21 (23%) were extenders. Nearly all (99%) were aware of Electronic-Florida Online Reporting of Controlled Substance Evaluation Program (EFORCSE) and 84% had registered accounts. More than 2/3 (73%) reported feeling pressured to prescribe opioids, and 70% reported receiving no formal education on identifying individuals at increased risk of opioid misuse. Approximately half (51%) reported that they used EFORCSE only when they suspect the patient may misuse the medication, 21% reported that they rarely used EFORCSE, and only 3% reported using PDMP every time that they prescribed opioids. Residents used PDMP less frequently than extenders and attending physicians. The most common barriers associated with PDMP use were related to access.

**Conclusions:**

Although most providers reported that they were aware of their states’ PDMP, utilization of the PDMP among emergency medicine providers in Florida remains low. Low utilization was associated with barriers to access. If further enhancements to PDMPs can be made to improve accessibility, then rates of PDMP utilization may increase.

**Electronic supplementary material:**

The online version of this article (doi:10.1186/s12245-017-0140-0) contains supplementary material, which is available to authorized users.

## Background

The prescription drug abuse epidemic is a rapidly growing public health concern that affects individuals of all ages. The overall prescription rate of opioids among Americans has significantly increased since 2000 [1]. The increasing rate in the prescription of opioids has been associated with a concomitant increase in opioid-related overdose deaths [2]. In 2014, the rate of death due to overdose was 1.5 times the number of deaths from motor vehicle collisions, with prescription opioids accounting for most of those deaths [3]. In 2012, enough opioids were prescribed by healthcare providers to provide every adult in the USA with a prescription of opioids to be taken daily for an entire month [4]. The over prescribing of opioids to individuals at high risk for misuse has contributed to this epidemic.

Although many physicians prescribe controlled substances, few are trained on how to appropriately identify individuals at risk for opioid abuse. A recent study found that most opioids are distributed by primary care physicians who were not trained appropriately in pain management or prescribing opioids while in residency or practice [5]. Unfortunately even fewer physicians received training while in medical school. Mezei et al conducted a survey of 117 American and Canadian medical schools to assess their respective pain curriculums. Of the 104 US medical schools involved in the study, only 10 reported including a required course on addiction. Compared to Canadian medical schools, their American counterparts spend approximately half the time training on pain management [6]. The physician’s lack of knowledge about an individual’s risk for opioid abuse may result in the providers unknowingly contributing to the opioid epidemic.

Pain is among the most common presentations to the emergency department, and opioids are commonly prescribed from the emergency department (ED) [7–9]. Studies have shown that emergency departments are frequently targeted by opioid abusers as a source for opioids, and as many as 88% of emergency physicians report having treated an individual at least once a week who was “doctor shopping” or displayed signs of aberrant behavior [10–12]. Pain is subjective, and it can be difficult to identify who is in pain and who is malingering in the emergency medicine setting. Studies have shown that ED physicians may be incorrect over a third of the time at recognizing individuals at risk for opioid abuse or misuse [13]. Unfortunately, with the lack of preexisting relationships with the patient, limited knowledge of opioid abuse and the limited time that the provider has with the patient in the emergency setting, it becomes difficult to identify individuals at risk for opioid abuse. While emergency medicine physicians usually prescribe small quantities of immediate release opioids to treat acute pain, they are often unaware that they may be contributing to the epidemic by providing overlapping prescriptions or unintentionally prescribing opioids to individuals at high risk of abuse.

Given that the emergency medicine setting may be a highly vulnerable one, the use of clinical tools that provide objective data regarding an individual’s risk of abusing opioids becomes imperative. It is recommended by the American College of Emergency Physician (ACEP) and the US Centers for Disease Control and Prevention that prior to prescribing opioids, the provider assess the individual patient’s risk for abuse [14, 15]. One common way to do this is to assess risk through the prescription drug monitoring program (PDMP).

Prescription drug monitoring programs were established to combat the growing prescription drug abuse epidemic. The PDMP provides clinicians with a report of the controlled substances that the patient has had prescribed to them and the prescribers. This allows for the provider to determine if the patient exhibits a pattern of behavior consistent with having a greater risk of opioid misuse or abuse. The PDMP was created in Florida in 2010. At the apex of the opioid epidemic, Florida was considered the epicenter of the opioid crisis. In a report from ARCOS, 90 of the nation’s top 100 purchasers of oxycodone were from practitioners in Florida [16]. In 2009, 1 in 8 deaths in Florida were related to prescription drug overdoses [17]. Florida’s PDMP has developed several outreach and educational events to increase awareness of and enhancements to the PDMP report to improve ease of use. Although studies have demonstrated that the PDMP has contributed to a decrease in oxycodone-related deaths in Florida, utilization among healthcare providers in Florida as in national assessments demonstrates suboptimal use of the Florida PDMP [18, 19].

This study further investigates the current utilization rate and perceptions of the Florida prescription drug monitoring programs among emergency medicine providers

## Methods

A 25-item anonymous survey was designed by the authors to assess utilization of Florida’s prescription drug monitoring program among emergency medicine providers in Florida. This PDMP is called the Electronic-Florida Online Reporting of Controlled Substance Evaluation Program (E-FORCSE). The survey was formatted using RedCap, a secure online Web application for building and managing online surveys and databases. A link to the survey was distributed within the Florida College of Emergency Physician’s (FCEP) weekly newsletter. FCEP is the state chapter of the national organization of the American College of Emergency Physicians, and its members include physicians, residents, nurse practitioners, and physician assistants who practice emergency medicine in the state of Florida. At the time of dissemination of the survey, there were approximately 1700 members of FCEP.

Participants who elected to participate in the survey were redirected to the electronic survey that began with University of Florida Institutional Review Board approved consent form. No identifiable information was collected, and the participant was allowed to end the survey at any time. There were no exclusion criteria.

The survey assessed the respondent’s comfort level with identifying drug seeking behavior, utilization of EFORCSE in their clinical practice, their perceptions of EFORCSE and barriers to EFORCSE. Some of the survey questions allowed the participant to select more than one response; therefore, for those questions, the response rate exceeded 100%. Branching logic was used where appropriate to reduce the total number of responses for some answers.

The survey was first published on the first page of FCEP’s weekly newsletter in December 2015. A link for the survey remained active for 5 weeks, closing the end of January 2016.

## Results

There were 396 views of the newsletter during the 5 week period. There were 88 total responses for the survey yielding a response rate of 22% of the views. Approximately two thirds (67%) of the responders were male. Over half (58%) of responders were 20–40 years of age. Physician assistants and nurse practitioners who completed the survey were characterized as extenders. Over half (62%) of responders were attending physicians, 13 (14%) were residents, and 21 (23%) were extenders. The majority of the respondents worked in academic medicine (60%) with an annual ED census over 75,000 (see Table 1).

**Table 1:** Demographics and characteristics of Florida emergency medicine healthcare providers that completed the survey (*N* = 90)

Male	60 (67%)
Age	
20–40 years of age	52 (58%)
41–50 years of age	21 (23%)
>50 years of age	17 (19%)
Practitioner type	
Attending physician	56 (62%)
Resident	13 (14%)
Extender	21 (23%)
Experience	
1–5 years	38 (42%)
>5–10 years	15 (17%)
>10 years	37 (41%)
Practice facility	
Academic	54 (60%)
Private or community	32 (36%)
Urgent care	4 (4%)
Average annual census	
<30 k	5 (6%)
30–75 k	33 (37%)
>75 k	52 (57%)

Although not shown nearly all (99%) respondents were aware of EFORCSE. While most (84%) respondents reported having registered accounts, only 2% reported that their employer required them to have one. More than two-thirds (70%) of providers reported having received no formal training in identifying individuals at risk for opioid abuse or misuse, and 73% reported feeling pressured to prescribe opioids to a patient. Though formal training was not required, 97% reported feeling comfortable identifying individuals at risk of opioid misuse in their practice.

When asked when providers would use EFORCSE, over half (51%) said “only when I suspect misuse or abuse.” Over 21% said they rarely used EFORCSE, 12% said they used it half of the time, and 9% said they never used EFORCSE when prescribing opioids. Only 3% of practitioners reported using EFORCSE every time that they prescribed opioids. When utilization was assessed by practitioner type as seen in Table 2, it was found that the attending physician and extender use of EFORCSE was similarly high (57 and 60%). Residents used EFORCSE less than both attending physicians and extenders with 50% of residents stating that they rarely use EFORCSE. Seventy-five percent of all responders stated that the results from EFORCSE altered their management less than 50% of the time.

**Table 2:** Utilization of EFORCSE characterized by practitioner type

	Attending physician (*N* = 57)	Resident (*N* = 12)	Extenders (*N* = 21)	Total (*N* = 90)
I do not use EFORCSE	4 (7%)	3 (25%)	1 (5%)	8 (9%)
I rarely use EFORCSE	10 (18%)	6 (50%)	3 (15%)	19 (21%)
I use EFORCSE only when I suspect misuse	32 (57%)	3 (25%)	12 (60%)	47 (51%)
I use EFORCSE most (>50%) of the time	9 (16%)	0	2 (10%)	11 (12%)
I use EFORCSE every time I prescribe opioids	1 (2%)	0	2 (10%)	3 (3%)

A total of 15 barriers were identified by providers (Table 3). The most common annoyance found with using EFORCSE being related to having to renew a password too frequently. When stratified by use of the system with high utilization being those who used the system more than 50% of the time when prescribing opioids, the most common barriers were system timing out too frequently and renewing a password too frequently. Among all other users the most common reason was the password renewal (68%) and a system that is too difficult to access (52%). Nearly half (45%) reported the process to acquire information from EFORCSE took too long (Table 3).

**Table 3:** The most commonly cited barriers to use of EFORCSE characterized by high utilizers (individuals that state they use EFORCSE greater than 50% of the time when prescribing opioids) and all others

High utilizers (*N* = 11)	All other users (*N* = 69)
The system times out too frequently (55%)	I have to renew my password too frequently (68%)
I have to renew my password too frequently (46%)	The system is too difficult to access (52%)
The process to acquire information from EFORCSE takes too long (27%)	The process to acquire information from EFORCSE takes too long (45%)
The information is not up to date (27%)	I forgot my password (39%)

Although not shown, most providers (81%) stated that EFORCSE was helpful in their daily practice and stated that the results of EFORCSE altered their management (85%). Greater than 90% of providers reported that the results of EFORCSE lead to interventions such as referring an at risk individual to pain management, referring an individual to substance abuse treatment, discussing the opioid use with other providers or the patient. Nearly all (94%) stated that they would utilize EFORCSE more in their daily practice if the barriers were removed and reported that they would prefer a single score that allowed easier interpretation of the PDMP data (76%). The data for this can be found in Additional file 1. 

## Discussion

As shown in this study, awareness of prescription drug monitoring programs is high among emergency medicine providers at all stages of training. Greater than 80% of survey participants reported that the prescription drug monitoring program is a useful tool in clinical practice and a similar amount reported that the results they received from the PDMP altered their management. Despite this, utilization remains suboptimal with approximately 85% of users stating that they utilize the tool less than 50% of the time when prescribing opioids. The lowest utilization was found in the most junior providers with residents or physicians in training reporting lower frequency of use than more experienced providers. This is important because it is the population that could benefit the most from the data.

Some have suggested that the PDMP be consulted prior to writing every opioid prescription [20]. The ideal utilization of the PDMP among emergency medicine physicians remains unclear. Currently, the state of Florida does not require mandatory use of the PDMP when prescribing opioids. From our survey, we note that most providers utilized PDMP only when the provider suspected that the individual would abuse or misuse the medication. While utilizing the PDMP every time a controlled substance is prescribed may not be feasible as it is currently constructed in a busy emergency department, only assessing the PDMP when the clinician suspects diversion will lead to a significant bias and limit the effectiveness of the tool. This behavior is directly influenced by the individual’s experiences, education, knowledge, and personal biases. The ED is an environment fraught with increasing demands and stressors which may make the lack of use of structured objective tools such as the PDMP more concerning in this vulnerable population. For example, a recent study demonstrated that as cognitive stressors commonly encountered in the emergency department such as fatigue, stress, time pressure, and complex decision making increase, practitioner’s unconscious biases may become amplified thus interfering with their care [21].

With a high rate of pain seen in the emergency department, patients expect to receive opioids upon discharge. In our study, 73% of providers reported that they felt pressured by the patient to prescribe opioids. This is concerning because patient satisfaction scores may negatively impact healthcare provider’s prescribing practices. Recently, there has been an increasing emphasis on linking patient satisfaction scores to reimbursement for hospitals and clinicians. Concern exist among some clinicians that not providing opioids from some patients may result in lower patient satisfaction scores [22, 23].

Despite the high rate of pain among individuals treated in the emergency department, we found only 30% of providers received any formal training in identifying individuals at risk for opioid misuse, which is concerning because studies have shown that identifying individuals at risk for opioid abuse can be difficult [13]. This can lead prescribers unknowingly contributing to the pain epidemic by prescribing individuals at increased risk for misuse of opioids. Given the high rate of potential abuse in this population and the limited information available for most ED providers at the time of the encounter, education on how to identify at risk individuals in the ED is imperative in combating this growing public health concern. Furthermore, the views and understanding of the risks of opioid therapy has significantly changed warranting continued education at all levels to ensure that clinicians are well informed on the safest manner to distribute opioids.

The ED encounter provides a unique opportunity to identify individuals at risk for substance abuse, so they can be linked to care. More than 90% of respondents reported that the information that they obtained from the PDMP led them to provide the individual with additional care related to addiction that ranged from counseling to providing information for substance abuse treatment facilities. Due to this population‘s limited access to care, the ED encounter may serve as the only opportunity for healthcare providers to intervene in this disease. The PDMP allows ED physicians to identify many individuals who would significantly benefit from receiving substance treatment who otherwise could go unrecognized.

Barriers to access were found to significantly reduce the utilization of PMDP within emergency departments. Greater than 90% of all survey participants reported that they would use the PDMP more if the barriers were removed. With the increasing ED census and limited time in the emergency department, implementing processes that are efficient is paramount. Barriers, particularly barriers to access, can significantly discourage utilization and disrupt the clinical workflow. If the barriers to access were remedied, it is reasonable to expect increased utilization.

There are several changes that could improve these barriers to access including decreasing the frequency of mandatory password changes, less frequent timeouts, and integration of the PDMP into the electronic medical record (EMR). Integration of the PDMP into the EMR not only would promote efficiency but make it feasible to consult the PDMP on every patient with minimal disruption in the clinical workflow. In a study conducted in Indiana, a PDMP system was integrated into the emergency department’s electronic medical record. The integration resulted in a significant increase in number of queries submitted to the PDMP. Nearly all (98%) of the clinicians reported that they preferred the integrated system [24]. By limiting barriers, we can increase its utilization thus facilitating safer prescribing habits of clinicians.

This is the first study to assess the perceptions of utilizations of emergency medicine providers in Florida regarding EFORCSE use after significant enhancements to Florida’s PDMP and outreach programs for providers to increase registration and utilization.

## Limitations

This study has potential limitations. The study only had 90 completed surveys and only included practitioners in Florida. Although this is a relatively small sample size, it included individuals from all regions of Florida and included a variety of practitioner types and practitioner settings.

## Conclusions

Although nearly all emergency medicine providers were aware of the Florida’s prescription drug monitoring program, use of this tool remains low in clinical practice. In our study, barriers to access were commonly listed as reasons for low utilization and few emergency medicine providers report receiving formal training in identifying individuals at risk for opioid misuse. Further research involving educating clinicians and integrating PDMP into the clinical practice is needed.

## Additional file

Additional file 1: The utilizaton and perceptions of the prescription drug monitoring program. (XLS 86 kb)

## Abbreviations

ACEP: American College of Emergency Physicians
ED: Emergency department
EFORCSE: Electronic-Florida Online Reporting of Controlled Substance Evaluation Program
EMR: Electronic medical record
FCEP: Florida College of Emergency Physicians
PDMP: Prescription drug monitoring program
US: United States

## References

[1] CDC. Wide-ranging online data for epidemiologic research (WONDER). Atlanta, GA: CDC, National Center for Health Statistics; 2016. Available at http://wonder.cdc.gov.

[2] Kuehn BM (2007). Opioid prescriptions soar: increase in legitimate use as well as abuse. JAMA.

[3] Centers for Disease and Prevention (CDC). (2016, January 1). Increases in Drug and Opioid Overdose Deaths - United States, 200-2014. MMWR. Morbidity and Mortality Weekly Reports. Available at https://www.cdc.gov/mmwr/preview/mmwrhtml/mm6450a3.htm. 10.15585/mmwr.mm6450a3 26720857

[4] Paulozzi L, Jones C, Mack K, Rudd R (2011). Centers for Disease Control and Prevention (CDC). Vital signs: overdoses of prescription opioid analgesics—United States, 1999–2008. MMWR Morb Mortal Wkly Rep.

[5] Jamison RN, Sheehan KA, Scanlan E, Matthews M, Ross EL (2014). Beliefs and attitudes about opioid prescribing and chronic pain management: survey of primary care providers. J Opioid Manag.

[6] Mezei L, Murinson B, Johns Hopkins Pain Curriculum Development Team (2011). Pain education in North American medical schools. J Pain.

[7] Tanabe P, Buschmann M (1999). A prospective study of ED pain management practices and the patients prospective. J Emerg Nurs.

[8] Todd KH, Ducharme J, Choiniere M, PEMI Study Group, et al. Pain in the emergency department: results of the Pain and Emergency Medicine Initiative (PEMI) multicenter study. J Pain. 2007;460–6. 10.1016/j.jpain.2006.12.005 17306626

[9] Hoppe JA, Nelson LS, Perrone J, Weiner SG (2015). Opioid prescribing in a cross section of US emergency departments. Ann Emerg Med.

[10] Wilsey BL, Fishman SM, Ogden C (2005). Prescription opioid abuse in the emergency department. J Law Med Ethics.

[11] Bachman JG, Johnston LD, O’Malley PM. Monitoring the future: questionnaire responses from the nation’s high school seniors. Survey Research Center, Institute for Social Research, University of Michigan, Ann Arbor, MI. Available at: http://monitoringthefuture.org/datavolumes/2010/2010dv.pdf.

[12] Shaffer EG, Moss AH (2010). Physicians’ perceptions of doctor shopping in West Virginia. WV Med J.

[13] Weiner SG, Griggs CA, Mitchell PM (2013). Clinician impression versus prescription drug monitoring program criteria in the assessment of drug-seeking behavior in the emergency department. Ann Emerg Med.

[14] Dowell D, Haegerich TM, Chou R (2016). CDC guideline for prescribing opioids for chronic pain—United States, 2016. JAMA.

[15] Cantrill, Stephen V, et al. "Clinical Policy: Critical Issues in the Prescribing of Opioids for Adult Patients in the Emergency Department." Annals of Emergency Medicine 60.4 (2012). 10.1016/j.annemergmed.2012.06.013 23010181

[16] Press release. Florida doctors no longer among the top oxycodone purchasers in the United States. Drug Enforcement Administration. Website. http://www.dea.gov/divisions/mia/2013/mia040513.shtml. Published April 5, 2013. Accessed 3 Jan 2016.

[17] Centers for Disease Control and Prevention (CDC). (2011, July 8). Drug overdose deaths—Florida, 2003–2009. MMWR. Morbidity and Mortality Weekly Reports, 60: 869–72. Website. https://www.cdc.gov/mmwr/preview/mmwrhtml/mm6026a1.htm. 21734633

[18] Delcher C, Wagenaar AC, Goldberger BA (2015). Abrupt decline in oxycodone-caused mortality after implementation of Florida’s prescription drug monitoring program. Drug Alcohol Depend.

[19] Electronic-Florida Online Reporting of Controlled Substances Evaluation 2014–2015 prescription drug monitoring program annual report. Florida Department of Health. Web site. http://www.floridahealth.gov/statistics-and-data/e-forcse/news-reports/_documents/2015-pdmp-annual-report.pdf. Published December 1, 2015. Accessed 6 Jan 2016.

[20] Listing of mandatory enrollment or query conditions. Prescription Drug Monitoring Program Training and Technical Assistance Center. Web site. http://www.pdmpassist.org/pdf/Mandatory_conditions.pdf. Published July 1, 2016. Accessed 13 July 2016.

[21] Johnson TJ, Hickey RW, Switzer GE (2016). The impact of cognitive stressors in the emergency department on physician implicit racial bias. Acad Emerg Med.

[22] Zgierska A, Miller M, Rabago D (2012). Patient satisfaction, prescription drug abuse, and potential unintended consequences. JAMA.

[23] Zgierska A, Rabago D, Miller M (2014). Impact of patient satisfaction ratings on physicians and clinical care. Patient preference and adherence.

[24] Abedtash H, Finnell JT (2013). A pilot study: integrating an emergency department with Indiana’s prescription drug monitoring program.

